# Determination of glycoside hydrolase specificities during hydrolysis of plant cell walls using glycome profiling

**DOI:** 10.1186/s13068-017-0703-6

**Published:** 2017-02-02

**Authors:** Johnnie A. Walker, Sivakumar Pattathil, Lai F. Bergeman, Emily T. Beebe, Kai Deng, Maryam Mirzai, Trent R. Northen, Michael G. Hahn, Brian G. Fox

**Affiliations:** 1 US Department of Energy Great Lakes Bioenergy Research Center, University of Wisconsin-Madison, Madison, WI 53706 USA; 2 Department of Biochemistry, University of Wisconsin-Madison, Madison, WI 53706 USA; 3 US Department of Energy Bioenergy Science Center, Oak Ridge National Laboratory, Oak Ridge, TN 37831 USA; 4 Complex Carbohydrate Research Center, University of Georgia, Athens, GA 30602 USA; 5 US Department of Energy Joint Bioenergy Institute, Emeryville, CA 94608 USA; 6 Sandia National Laboratories, Livermore, CA 94551 USA; 7 Lawrence Berkeley National Laboratory, Berkeley, CA 94720 USA

**Keywords:** Glycoside hydrolase, Xylanase, Xyloglucanase, Glycome profiling, Nanostructure-initiator mass spectrometry, Enzyme specificity

## Abstract

**Background:**

Glycoside hydrolases (GHs) are enzymes that hydrolyze polysaccharides into simple sugars. To better understand the specificity of enzyme hydrolysis within the complex matrix of polysaccharides found in the plant cell wall, we studied the reactions of individual enzymes using glycome profiling, where a comprehensive collection of cell wall glycan-directed monoclonal antibodies are used to detect polysaccharide epitopes remaining in the walls after enzyme treatment and quantitative nanostructure initiator mass spectrometry (oxime-NIMS) to determine soluble sugar products of their reactions.

**Results:**

Single, purified enzymes from the GH5_4, GH10, and GH11 families of glycoside hydrolases hydrolyzed hemicelluloses as evidenced by the loss of specific epitopes from the glycome profiles in enzyme-treated plant biomass. The glycome profiling data were further substantiated by oxime-NIMS, which identified hexose products from hydrolysis of cellulose, and pentose-only and mixed hexose-pentose products from the hydrolysis of hemicelluloses. The GH10 enzyme proved to be reactive with the broadest diversity of xylose-backbone polysaccharide epitopes, but was incapable of reacting with glucose-backbone polysaccharides. In contrast, the GH5 and GH11 enzymes studied here showed the ability to react with both glucose- and xylose-backbone polysaccharides.

**Conclusions:**

The identification of enzyme specificity for a wide diversity of polysaccharide structures provided by glycome profiling, and the correlated identification of soluble oligosaccharide hydrolysis products provided by oxime-NIMS, offers a unique combination to understand the hydrolytic capabilities and constraints of individual enzymes as they interact with plant biomass.

**Electronic supplementary material:**

The online version of this article (doi:10.1186/s13068-017-0703-6) contains supplementary material, which is available to authorized users.

## Background

The cell walls of plants provide renewable material that can be converted to energy (biofuels) and chemicals (biocommodities). Effective deployment of biofuels can help to reduce greenhouse gas emissions [1]. Moreover, supplementation of petroleum-based energy and chemicals with renewable substitutes can increase national and economic security during times of fluctuating oil price and availability. Some ideal sources of plant biomass for energy and biocommodities production include agricultural wastes (e.g., corn stover and wood) and low-input, high-yield perennial plants (e.g., switchgrass or poplar) that can potentially be grown on marginal lands [2].

Plant biomass is composed of polymers such as lignin, cellulose, hemicellulose, pectin, and other polysaccharides. In grasses like corn stover and switchgrass, lignin accounts for 10–20% of dry weight, cellulose 35–45%, hemicellulose 20–40%, and pectin at lower percentage [3–7]. The ultrastructure of plant cell walls consists of bundles of crystalline cellulose microfibrils that are non-covalently associated with the various hemicellulose polysaccharides including xyloglucans, while glucuronoarabinoxylans are thought to provide both covalent and non-covalent connections between interwoven strands of cellulose, xyloglucan, and pectin [8–15]. Connections between xylan and pectin involving cell wall proteins were also identified recently [16, 17]. Consequently, plant biomass is a durable material that is difficult to efficiently convert into simple sugars and monomeric aromatic compounds.

This work focuses on the hemicelluloses, which include xylans, arabinoxylans, and xyloglucans. Xylans consist of a β-1,4-linked d-xylose backbone that can have a variety of substituents [18, 19]. Glucuronoarabinoxylans (heteroxylans), which are abundant in grasses, have primary glucuronyl substitutions at the C(O)2 and/or arabinofuranosyl substitutions at C(O)3 positions [18, 20, 21]. These primary substitutions may in turn have glucuronosyl, methyl-glucuronosyl, or feruloyl substitutions, which introduces additional variation in xylan structure and also provides opportunities for crosslinking of xylan to lignin [22–24]. Xyloglucans found in grasses have a β-1,4-linked d-glucose backbone with a regular pattern of α-1,6 xylose branching [25]. The branches can have galactose and/or arabinose substituents, and the galactose can also be fucosylated at C(O)2, though the latter modification is not highly prevalent in monocot grasses [26]. Other polysaccharides in grasses include mixed-linkage β-glucans [18, 20, 21], mannans [27, 28], and pectins [9, 14, 29].

Pretreatments increase the efficiency of enzymatic hydrolysis of plant biomass. These include acidic (including organosolv and ionic liquids, IL, USA), neutral/water, and alkaline pretreatments [20, 30–32]. One alkaline pretreatment, ammonia fiber expansion (AFEX), exposes plant biomass to anhydrous ammonia gas at high pressure for a short time [33]. Rapid depressurization leads to explosive expansion of the entrained ammonia gas, which physically disrupts the plant cell wall [20, 30, 34–36]. In the AFEX process, base-labile acetyl and feruloyl esters that modify and/or crosslink different polysaccharides in the cell wall are cleaved by ammonolysis to amides [35–37]. Relevant to this study, glycome profiling and other studies suggest that AFEX apparently causes relatively few other changes in the covalent bonding present in the hemicellulose and cellulose fractions [32, 33, 37, 38].

In nature, many different families of glycoside hydrolases (GHs) cleave biomass polysaccharide chains into oligo- and monosaccharides [39]. Cellulases are subdivided into endo- and exo-glucanases and β-glucosidases according to their ability to hydrolyze glycosidic bonds in cellulose and β-glucan polymers internally or at chain ends, or to hydrolyze glucose from cellobiose and other short, soluble oligosaccharides, respectively. Xylanases (endo-xylanases and β-xylosidases) and mannanases (endo-mannanases and β-mannosidases) hydrolyze the glycosidic bonds of xylans and mannans, respectively, with similar functional classifications. Other accessory enzymes such as α-glucuronidase and α-l-arabinofuranosidase are utilized to release branching substituents from the backbone chains; in these specific examples, from the backbone of xylans. Enzymes capable of these reactions fall into many different GH families [6, 40–43].

In this work, we compare reactions of three enzymes from *Ruminiclostridium thermocellum* using two AFEX-pretreated grasses (corn stover and switchgrass) as the substrates. Two complementary techniques, glycome profiling and oxime-nanostructure initiator mass spectrometry (oxime-NIMS), have been used in this work. Glycome profiling uses a large and diverse suite of monoclonal antibodies (mAbs) to detect most major non-cellulosic polysaccharide epitopes present in the plant cell walls, including those in hemicelluloses [44, 45]. Glycome profiling has been used previously to reveal modifications in plant cell walls after diverse pretreatment processes [37, 46, 47], but these previous studies have not sought to explicitly link the impact of single enzymes on cell wall hydrolysis. The work reported here demonstrates that the reactions of individual GH enzymes with intact plant biomass can be studied effectively using glycome profiling. Our analyses revealed differences in the specificities of individual, purified enzymes in their reactions with AFEX-pretreated grass biomass samples.

Oxime-NIMS is another technique with great utility in studies of the hydrolysis of plant biomass [48]. This method allows quantitative, high sensitivity detection of enzyme-solubilized reducing sugars and oligosaccharides, and assignment of the proportion of hexose and pentose sugars present. Oxime-NIMS has also proven useful in elucidating differences in the behavior of different enzymes in their reactions with pure oligosaccharides and pretreated plant biomass [48–51]. Oxime-NIMS carried out in the current work revealed diagnostic differences in the soluble products released by the activities of three different purified enzymes with plant cell walls.

This combination of approaches provides new understanding of the activities of GH enzymes on the polysaccharide fraction of grass cell walls. The specificities for cell wall epitopes identified and, conversely, the specificities lacking in the three enzymes studied offer potential to guide the improvement of simple combinations of enzymes for cell wall hydrolysis.

## Results

### Enzymes studied

Three enzymes from *Ruminiclostridium thermocellum* have been investigated. One of these, the GH5 catalytic domain of CelE (Cthe_0797), abbreviated CMX00, is a broad specificity enzyme that can hydrolyze cellulose, mannans, and xylans [48, 52]. To increase the reactivity with insoluble polysaccharides, the CelE catalytic domain was fused to the carbohydrate binding module CBM3a from the cellulosome scaffoldin of *R. thermocellum* [48]. The fused enzyme, CMX00_3a, reacted with both the cellulose and hemicellulose fractions of pretreated plant biomass, particularly IL-treated biomass [48, 50].

The other two enzymes studied here are xylanases: XynY (Cthe_0912) and XynA (Cthe_2972) [53, 54]. XynY, containing a GH10 catalytic domain, was more reactive with the xylan fraction of IL-treated switchgrass than CMX00_3a [48], but did not react with cellulose. In addition, XynA, a GH11 xylanase, was of interest because of possible distinctions in the enzymatic capabilities of the xylanase members of GH10 and GH11 families [42, 53, 55–58].

**Fig. 1:**
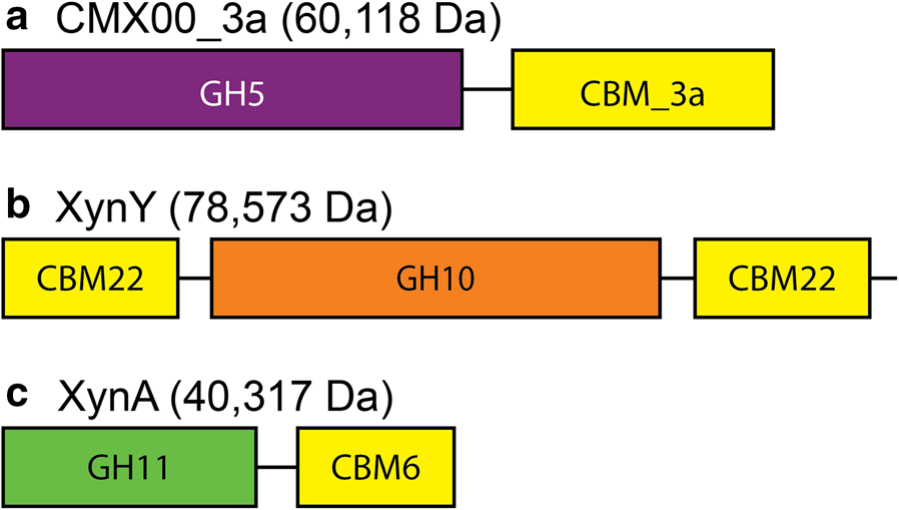
Schematic representations of the arrangement and relative sizes of the domains of the three enzymes studied. The N termini of the enzymes are on the *left*. *Lines* between domains represent linker peptides. The GH5 domain of CMX00_3a is colored* purple*; GH10 domain of XynY is colored* orange*; and the GH11 domain of XynA is colored *green*. Different CBM domains are colored *yellow*. The molecular weight of each enzyme is indicated

Figure 1 provides a schematic representation of the domain structures of the enzymes used in this study. CMX00_3a (Fig. 1) consists of the GH5 catalytic domain (codons 36-388) from *R. thermocellum* Cthe_0797 (CMX00) connected to the CBM3a domain (codons 323-523) from *R. thermocellum* scaffoldin, Cthe_3077 [48] using an interdomain linker from Cthe_3077 (codons 324-363). The molecular mass of CMX00_3a is 60,118 Da. CMX00 hydrolyzes cellulose, mannan, and xylan, and so is abbreviated CMX, with improved activity with different insoluble polysaccharides given by fusion to a CBM with appropriate binding specificity [48, 50]. The structural basis of CBM3a binding to crystalline cellulose is well established [59].

The XynY construct used in this work (Fig. 1) consists of the GH10 catalytic domain and the CBM22 domains found on each side of the catalytic domain in Cthe_0912 (codons 34-720), and has a molecular mass of 78,573 Da. Relative to the natural enzyme [54], this construct removes the signal peptide from the N-terminus, and the dockerin and esterase domains from the C-terminus. CBM22 has preference for binding to xylans [50, 60–62]. XynY has been observed to be specific for reaction with xylan-containing (β-1,4 linked xylose) substrates ([48] and see below).

The XynA construct (Fig. 1) used here consists of the GH11 and CBM6 domains (codons 29-375), and has a molecular mass of 40,317 Da. Relative to the natural enzyme (Cthe_2972) [53], sequences for the signal peptide, dockerin, and polysaccharide deacetylase domains were removed. CBM6 is described to have binding preference for a variety of polysaccharides including β-1,4-glucan, lichenan, arabinoxylans, and xylans [50, 63]. XynA has been reported to be specific for reaction with xylan-containing (β-1,4 linked xylose) substrates [53], but not with xyloglucan (β-1,4 linked glucose with α-1,6 xylose branches). This latter reactivity is demonstrated below.

### Enzyme reactions with pure substrates and untreated biomass

**Fig. 2:**
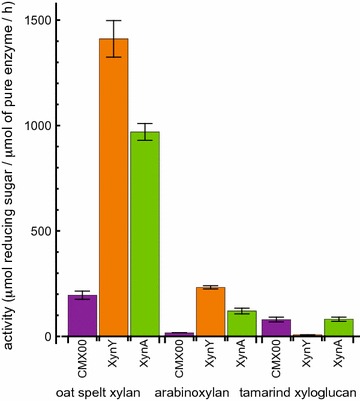
Enzyme reactions with isolated polysaccharide substrates oat spelt xylan, wheat arabinoxylan, and tamarind xyloglucan. Activities detected are reported as µmol of total reducing sugar released per µmol of pure enzyme per h at 55 °C; results for oat spelt xylan and wheat arabinoxylan are from oxime-NIMS, results for tamarind xyloglucan are from DNS. The *color bars* depict the different enzymes used: CMX00_3a (*purple*); XynY (*orange*); XynA (*green*).* Error bars* represent the standard deviation of reactions carried out in triplicate

CMX00_3a, XynY and XynA were reacted with isolated hemicellulosic polysaccharides to identify differences in specific activities and reaction specificities (Table 1; Fig. 2). We used oxime-NIMS to identify individual products from enzyme reactions with isolated hemicellulosic polysaccharides (Table 1). XynY had the highest enzyme activity followed by XynA, with a soluble product distributions dominated by pentose (X1) and pentobiose (X2). Oxime-NIMS showed that CMX00_3a had about 15–25% of the activity with oat spelt xylan relative to XynY and XynA, but the dominant products were shifted toward pentobiose (X2), pentotriose (X3), and pentotetraose (X4). All three enzymes had considerably lower activity with wheat arabinoxylan (Fig. 2), and again XynY was the most reactive, while CMX00_3a was only ~1% as reactive.

**Table 1:** Soluble oligosaccharide products detected by oxime-NIMS from hydrolysis of purified polysaccharides

	P1^a^	P2	P3	P4	P5	Total	Average	Stdev
Oat spelt xylan								
CMX00_CBM3a	2	27	43	28	14	113	125	13
	2	28	49	31	14	124		
	2	29	48	40	19	139		
XynY	225	403	114	68	15	826	855	37
	236	455	110	83	13	896		
	216	420	104	88	13	842		
XynA	88	277	2	5	118	488	468	25
	80	285	4	4	104	477		
	80	260	3	5	93	440		

	P1^b^	P2	P3	P4	P5	Total	Average	Stdev
Arabinoxylan								
CMX00_CBM3a	1	1	0	0	0	1	1	0
	1	0	0	0	0	1		
	1	0	0	0	0	1		
XynY	34	87	20	26	21	188	200	16
	39	102	24	29	24	219		
	36	90	20	25	22	193		
XynA	16	30	0	3	11	61	62	2
	17	34	0	3	11	65		
	16	32	0	2	11	61		

Additional abbreviations used: *P1* pentose (i.e., likely xylose), *P2* pentobiose, *P3* pentotriose, *P4* pentotetraose, *P5* pentopentose, with ~70:15:10 composition of xylose:glucose:arabinose present. No mass signatures for hexose sugars were identified

^a^Activity reported as µmol of reducing sugar released per hour per µmol of purified enzyme determined by oxime-NIMS as described in “Methods” section

^b^ *P1* pentose (xylose and arabinose will give rise to the same mass signature in oxime-NIMS; with a 38:62 composition of arabinose and xylose and less than 1% of glucose, galactose, and mannose present; no mass signatures for hexose sugars were identified), *P2* pentobiose, *P3* pentotriose, *P4* pentotetraose, *P5* pentopentose

Although DNS indicated both CMX00_3a and XynA released reducing sugars from tamarind xyloglucan (shown in Fig. 2), no small soluble products were detected by oxime-NIMS. Tamarind seed xyloglucan consists of a cellulosic backbone (β-1,4 linked glucose), where, on average, 3 out of 4 glucosyl backbone residues have xylose substituents, making this a relatively heavily branched substrate [64]. The xylosyl residues can be further extended with galactosyl groups [64]. This high degree of substitution along the glucan backbone led to reducing sugar ends that could be detected by DNS, but these products were not sufficiently small to be soluble and detected by oxime-NIMS. In support of this, MALDI-MS showed CMX00_3a and XynA produced oligosaccharides with compositions of Glc_4_-Xyl_3_ (*m/z* = 1085, M + Na), Glc_5_-Xyl_4_ (*m/z* = 1247, M + Na) and Glc_6_-Xyl_5_ (*m/z* = 1409, M + Na). These products were not observed from reactions with XynY, also consistent with the DNS results.

### Pretreatment-induced changes in plant biomass glycans

AFEX pretreatment is effective on grasses [37, 65], and causes minimal changes in the hemicellulose content [66]. AFEX leads to ammonolysis of ester bonds in plant cells walls, for example, acetyl esters of xylan and feruloyl esters that crosslink arabinoxylan strands to each other and to lignin are susceptible, leading to the production of acetamide and feruloylamide [36]. As previously identified for hydrothermal pretreatment [67], most other bond types in hemicellulose are less affected by AFEX, leading to overall high-yield retention of the hemicellulose fraction [30]. This retention makes AFEX a good match for pairing with polysaccharide-epitope sensitive techniques like glycome profiling.

**Fig. 3:**
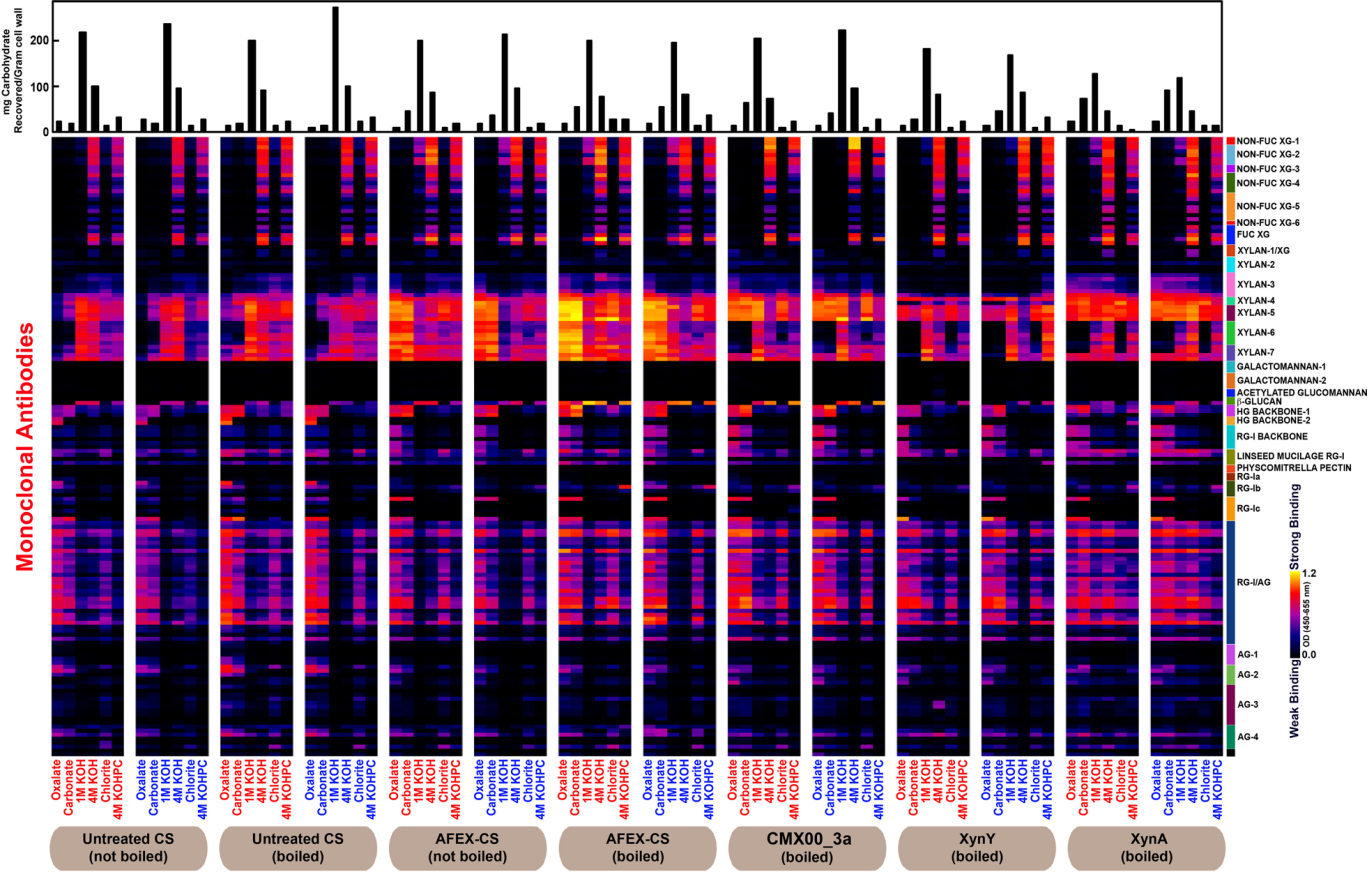
Glycome profiles obtained from successive extractions of the solid fractions remaining after reactions with corn stover (CS) indicated at the bottom of the figure. From *left to right*, the solid fractions are from samples of untreated CS (not boiled and boiled), AFEX-CS (not boiled and boiled), and AFEX-CS reacted with either CMX00_3a, XynY or XynA. Enzyme digestion reactions were incubated for 24 h at 55 °C and included 1 g of substrate in a 10-mL reaction with either no enzyme, 0.3 µmol of CMX00_3a, 0.1 µmol of XynY, or 0.2 µmol of XynA as indicated. All enzyme reactions were boiled to stop the enzyme reaction. The total carbohydrate extracted at each step of the glycome profiling workup is shown as the *bar graph* at the *top of the figure*. Each extract was loaded at 300 ng glucose equivalents/well on the ELISA plates. The glycan binding specificities of different antibody clades are shown at the *right* [45], with the following abbreviations: *XG* xyloglucan, *HG* homogalacturonan, *RG* rhamnogalacturonan, and *AG* arabinogalactan. Further information on the binding specificities of the antibodies is provided in the Additional file 1. The color scheme of the heatmaps depicts mAb binding strengths with *dark blue* representing no binding, *red* intermediate, and *bright yellow* strongest binding

**Fig. 4:**
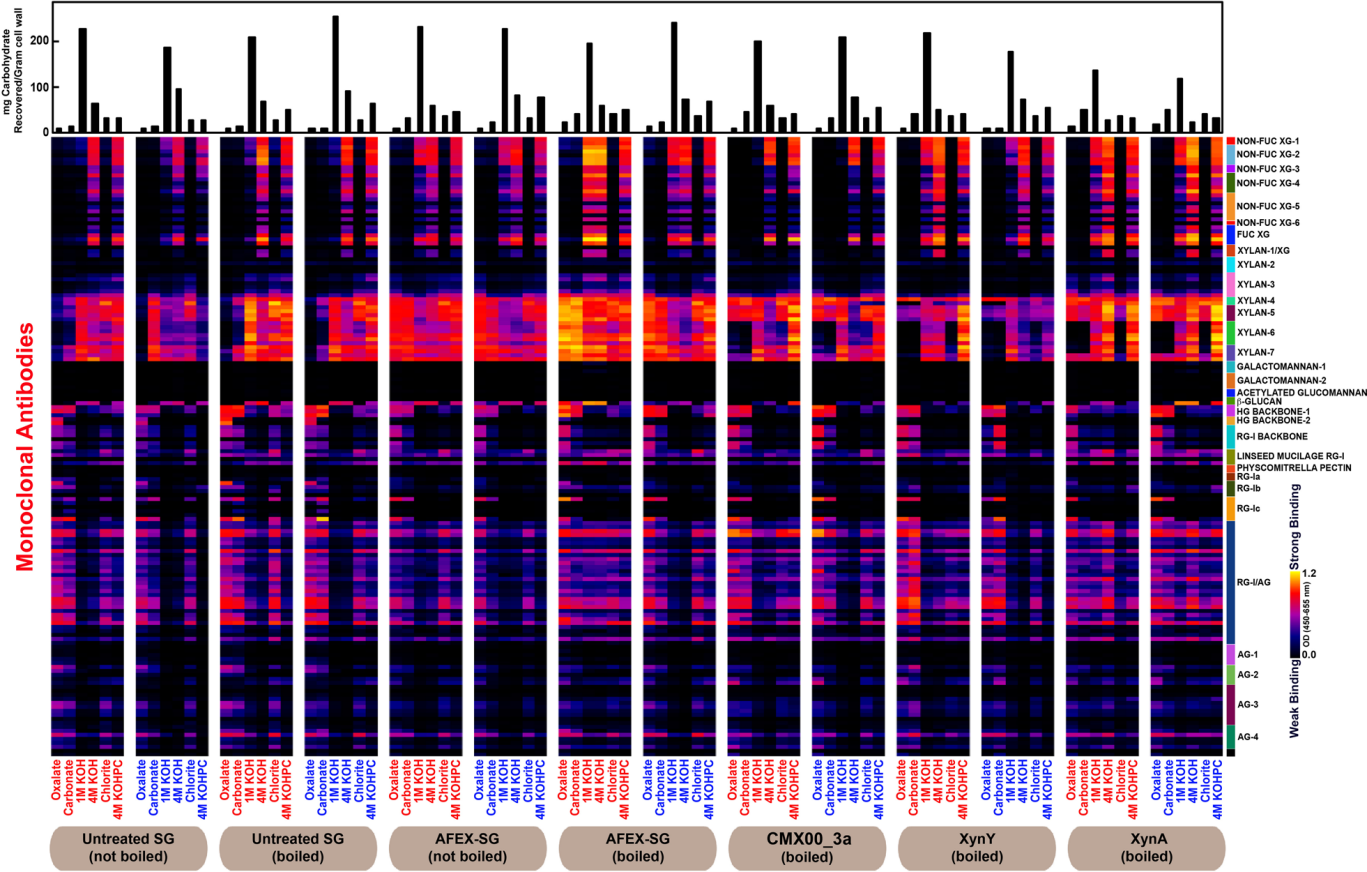
Glycome profiles obtained from successive extractions of the solid fractions remaining after reactions with switchgrass (SG) indicated at the *bottom of the figure*. From *left to right*, the solid fractions are from samples of untreated SG (not boiled and boiled), AFEX-SG (not boiled and boiled), and AFEX-SG reacted with either CMX00_3a, XynY or XynA. Enzyme digestion reactions were carried out as in Fig. 3. Enzyme reactions were boiled to inactivate the enzyme. The total carbohydrate extracted at each step of the glycome profiling workup is shown as the *bar graph* at the *top of the figure*. Each extract was loaded at 300 ng glucose equivalents/well on the ELISA plates. The glycan binding specificities of different antibody clades are shown at the right [45], with the following abbreviations: *XG* xyloglucan, *HG* homogalacturonan, *RG* rhamnogalacturonan, and *AG* arabinogalactan. Further information on the binding specificities of the antibodies is provided in the Additional file 1: Table S1. The color scheme of the heatmaps depicts mAb binding strengths with *dark blue* representing no binding, *red* intermediate, and *bright yellow* strongest binding

Figures 3 and 4 show glycome profiles of corn stover (CS) and switchgrass (SG), respectively, that were either untreated or AFEX-treated. The AFEX-treated biomasses were also incubated with different enzymes. Since enzymatic incubation was terminated by boiling the enzyme-biomass mixture, we also included separate untreated and AFEX-treated controls (incubated with buffer) that were boiled along with corresponding not-boiled controls. Results from duplicate experiments in Figs. 3 and 4 are shown for each plant substrate, side by side. The amount of carbohydrate released at each extraction step (given in the bar graphs at the top of Figs. 3 and 4 and also in Table 2), and the amount of antibody binding to the extracted polysaccharides (indicated by the color intensity in the glycome profiles) are shown. The identities of the antibody classes are shown at the right, and further information on these antibodies is provided in Table S1 of the Additional file 1. Overall, across all biomass samples analyzed, the replicate glycome profiles were nearly identical. Furthermore, comparison of glycome profiles of “not-boiled” versus “boiled” untreated CS and SG biomass samples showed only subtle variations suggesting minimal or insignificant alteration caused by boiling on the overall extractability of cell wall carbohydrates in untreated plant biomass. In accord with earlier studies [37, 38], AFEX pretreatment leads to an enhanced extractability of non-cellulosic cell wall polysaccharides in both CS and SG. For instance, in both AFEX-pretreated CS and SG, an enhanced abundance of epitopes from unsubstituted and substituted xylans (recognized by XYLAN-3, -4, -5, -6, and -7 groups of mAbs) was observed in the least harsh extracts (oxalate and carbonate). Further, enhanced extractability of xyloglucan epitopes (recognized by XG-1, XG-2, and FUC-XG groups of mAbs) was evident in the 1 M KOH extracts of AFEX-treated CS and SG samples. When boiled, AFEX-treated CS and SG samples showed slightly enhanced extractability of xylans, pectin, and mixed linkage glucan epitopes in the oxalate and/or carbonate extracts, as indicated by the enhanced abundance of epitopes detected by the XYLAN-4 through XYLAN-7 groups of mAbs, the rhamnogalacturonan-I backbone group of mAbs (in both oxalate and carbonate extracts of CS and subtle increase in oxalate extract of SG) and β-GLUCAN directed antibody, BG-1.

**Table 2:** Amount of carbohydrate extracted during glycome profiling per gram of cell wall

	Untreated	Boiled	AFEX	AFEX, boiled	CMX00	XynY	XynA
Corn stover							
Oxalate	2.3 ± 0.0	5.9 ± 0.0	5.0 ± 1.1	8.6 ± 1.4	4.1 ± 0.1	3.9 ± 0.1	9.6 ± 0.9
Carbonate	3.2 ± 0.2	4.4 ± 0.1	5.1 ± 0.5	4.6 ± 0.4	4.4 ± 0.8	4.0 ± 2.0	9.3 ± 0.6
1 M KOH	81.4 ± 1.5	61.4 ± 1.9	48.7 ± 1.8	46.4 ± 0.2	31.9 ± 0.9	21.1 ± 0.1	20.2 ± 1.4
4 M KOH	44.5 ± 2.1	26.3 ± 1.4	37.4 ± 1.8	32.7 ± 7.9	21.6 ± 2.6	11.1 ± 1.7	18.7 ± 0.2
Chlorite	7.0 ± 1.3	5.8 ± 0.4	3.6 ± 0.4	5.8 ± 1.3	2.2 ± 0.1	3.4 ± 0.6	2.9 ± 0.1
4 M KOHPC	9.4 ± 0.8	7.1 ± 2.9	7.8 ± 1.0	8.6 ± 2.8	4.2 ± 0.7	6.8 ± 3.5	3.2 ± 1.1
Total solid^a^	147.9	111.0	107.5	106.8	68.4	50.3	63.9
Soluble^b^	1.5	1.5	1.5	1.5	44.6	65.5	58.9
Total^c^	149.4	112.5	109.0	108.3	113.1	115.8	122.8
Switchgrass							
Oxalate	1.7 ± 0.5	1.8 ± 0.7	3.4 ± 0.5	5.5^d^	3.2 ± 0.0	1.5 ± 0.6	6.5 ± 1.1
Carbonate	2.5 ± 1.2	2.2 ± 0.3	3.4 ± 0.7	3.2^d^	2.6 ± 0.1	3.6 ± 1.6	4.8 ± 0.2
1 M KOH	58.4 ± 13.1	63.9 ± 13.3	30.6 ± 10.0	64.4^d^	33.8 ± 13.5	13.9 ± 1.0	30.6 ± 7.6
4 M KOH	20. ± 5.1	37.9 ± 9.0	14.0 ± 8.3	19.8^d^	13.7 ± 2.4	9.0 ± 2.0	8.8 ± 4.2
Chlorite	7.2 ± 0.4	7.0 ± 0.7	8.3 ± 1.3	6.8^d^	5.7 ± 0.7	9.3 ± 3.3	7.1 ± 1.1
4 M KOHPC	10.8 ± 12.0	29.8 ± 5.7	18.5 ± 1.8	23.8^d^	15.6 ± 1.4	8.1 ± 0.5	14.2 ± .1
Total solid^a^	100.5	142.5	78.2	123.3^d^	74.5	45.4	72.1
Soluble^b^	0.6	0.6	0.6	0.6^d^	46.3	69.8	70.0
Total^c^	101.1	143.1	78.8	123.9^d^	120.9	115.2	142.1

^a^Reducing sugar equivalents extracted from the solid fraction and quantified as mg quantities as reported elsewhere [46, 47]. Standard deviation calculated from two experimental replicates carried through the entire analysis method

^b^Reducing sugar equivalents present in the soluble fraction determined by oxime-NIMS (mg)

^c^Sum of solid and soluble fractions

^d^For boiled, AFEX-pretreated switchgrass, only single reactions were performed

**Fig. 5:**
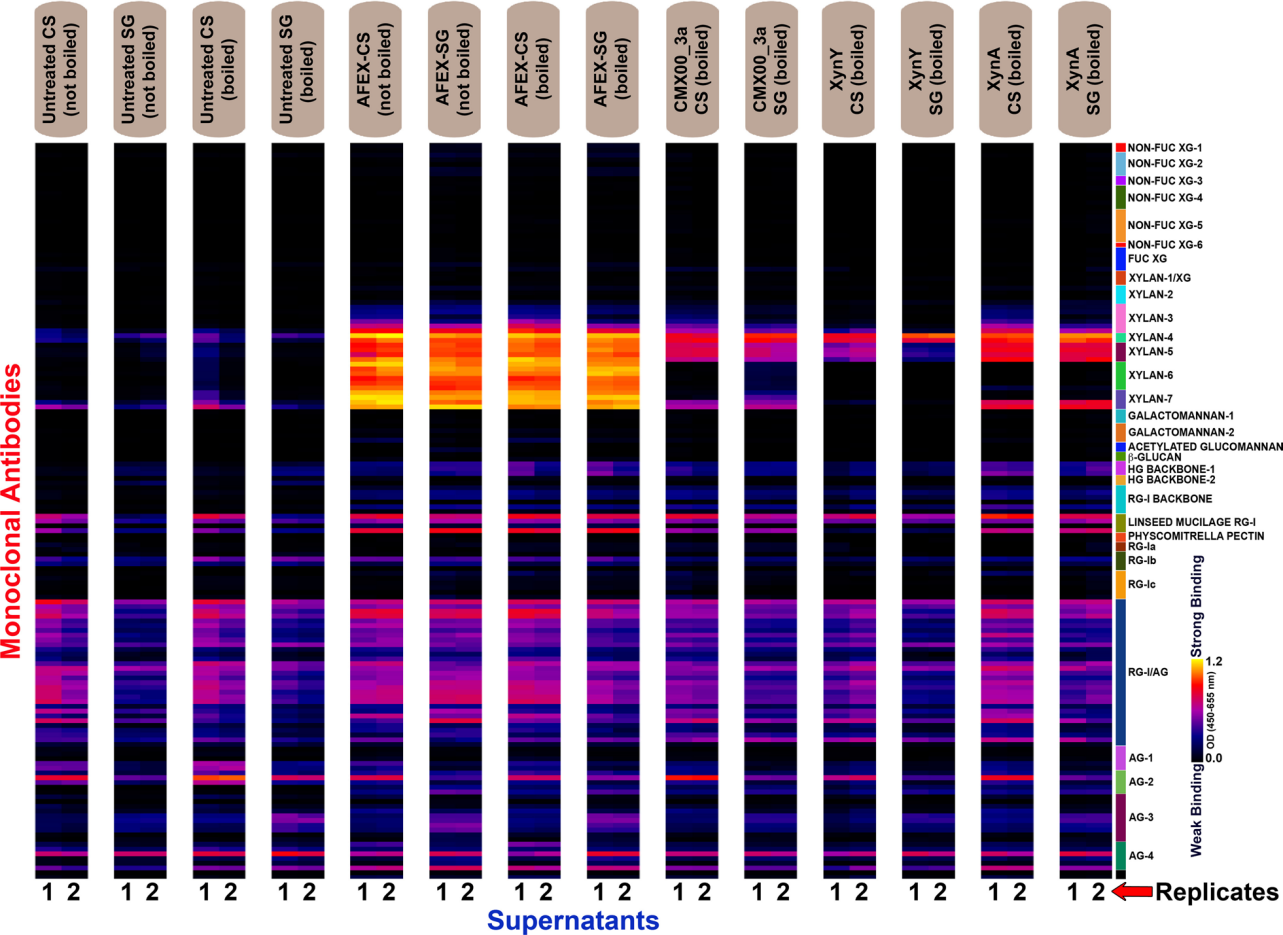
Antibody screening of the supernatants of controls and enzyme reactions. The various sample preparations are indicated at the *top of the figure*. Supernatants were applied to the ELISA plates (300 ng glucose equivalents/well) and allowed to dry overnight. ELISAs were carried out on replicates 1 and 2 in the same way as those done in the glycome profiling experiments described in Fig. 3. The glycan binding specificities of different antibody clades are shown at the right [45], with the following abbreviations: *XG* xyloglucan, *HG* homogalacturonan, *RG* rhamnogalacturonan, and *AG* arabinogalactan. Further information on the binding specificities of the antibodies is provided in the Additional file 1: Table S1. The color scheme of the heatmaps depicts mAb binding strengths with *dark blue* representing no binding, *red* intermediate, and *bright yellow* strongest binding

Figure 5 shows the ELISA-based screening of the supernatant fractions from controls and enzyme treatments where the entire suite of cell wall glycan-directed mAbs used for glycome profiling was employed. These fractions contain soluble cell wall polysaccharides that were (1) endogenously present in the biomass, (2) released by the AFEX pretreatment, or (3) released and/or modified by enzyme action. In the untreated, unboiled CS and SG supernatants, pectic arabinogalactan epitopes were present as recognized by mAbs belonging to the linseed mucilage RG-I, RG-Ib, RG-1/AG, and AG-1 through AG-4 groups. Traces of xylan epitopes were also noted in these fractions. Boiling of either of the supernatants from untreated biomass resulted in a significant loss of these signals, probably because the glycans are attached to protein backbones that precipitate and are lost as a consequence of boiling. In contrast, supernatants from AFEX-treated CS and SG had substantial presence of unsubstituted and substituted xylans as indicated by increased binding by the XYLAN-3, -4, -5, -6, and -7 group antibodies, in addition to the arabinogalactan epitopes present in the controls. Xyloglucan epitopes were not detected in any of the soluble fractions analyzed.

### Hydrolysis of biomass by three xylan-directed enzymes

We examined the activities of individual enzymes by incubating them with AFEX-treated CS and SG biomasses and subsequently conducting glycome profiling analyses of the unhydrolyzed insoluble residues. The presence of enzymes decreased the amount of total carbohydrate extracted in each glycome profile cell wall extract prepared from the residual solids (Table 2). Table 2 also shows that there is good mass balance when accounting for the sum of carbohydrate in the solid and soluble fractions, particularly for the CS experiment (~10% relative difference in total). The specificities of the individual purified enzymes during biomass hydrolysis were revealed by the reduced or completely abolished binding of specific mAbs to one or more of the sequential extracts prepared from the residual unhydrolyzed solids. In general, the glycome profiles of the residual solids remaining after enzyme treatment were different for each of the three enzymes studies here, with some overlap of reactivity.

### Effects of CMX00_3a activity

Compared to AFEX-treated CS and -SG not exposed to enzyme, the glycome profiles of the unhydrolyzed residual solids left after reaction with CMX00_3a (GH5_4) showed major changes. These changes included a near complete loss of epitopes detected by the XYLAN-6 groups of antibodies (Figs. 3, 4) in the oxalate, carbonate, 4 M KOH and chlorite extracts. In oxalate and carbonate extracts, there was a significant loss of xylan epitopes detected by the XYLAN-7 group of mAbs with the exception of two mAbs in this group, CCRC-M152 and CCRC-M149, especially in the case of SG. There was also a reduction in the xylan epitopes recognized by the XYLAN-3 group of mAbs. In addition to the above effects of this enzyme on xylans, there was a complete loss of xyloglucan epitopes in the 1 M KOH extracts of unhydrolyzed residues from both CS and SG as indicated by the absence of binding of various NON-FUC XG mAbs and FUC-XG mAbs to these extracts.

The supernatant fractions of AFEX-CS and SG residues treated with CMX00_3a (Fig. 5) contained xylan epitopes that were detected by some xylan-directed antibodies, particularly those from the XYLAN-5 (methyl GlcA-substituted xylan) and some mAbs of the XYLAN-7 group (CCRC-M152 and CCRC-M149, homoxylan epitopes with degree of polymerization >3). However, all other xylan epitopes were absent compared to the supernatants from AFEX-treated control biomass. This suggests the presence of xylan polymers released to the supernatants by AFEX pretreatment that are resistant to hydrolysis by CMX00_3a. There was also a slight reduction in the overall abundance of various other cell wall glycan epitopes detected in the enzyme-treated supernatants, in general, relative to the supernatants from AFEX-treated biomass (e.g., RG-1/AG and AG epitopes). CMX00_3a can hydrolyze soluble oligosaccharides into shorter pieces, typically-biose and -triose fragments [48, 51], and these molecules are too small to be detected by the ELISA (small molecules do not adhere to the wells of the plates used for the assays), likely accounting for the loss in detected intensity in the soluble fraction. However, these smaller molecules were readily detected by oxime-NIMS, and those results are presented below.

### Effects of XynY activity

The glycome profiles of AFEX-treated CS and SG residues treated with XynY (GH10) were distinct from the profiles obtained with CMX00_3a. Both XynY and CMX00_3a were effective in removing the xylan epitopes that are recognized by the XYLAN-6 group of mAbs from oxalate, carbonate and chlorite extracts, but CMX00_3a also removed more of the XYLAN-6 detected epitopes from the 4 M KOH extracts than did XynY. XynY completely eliminated epitopes detected by the XYLAN-3 antibodies (where the structure(s) of the epitope are unknown), and was more effective than either CMX00_3a or XynA in removing xylan epitopes that are detected by the XYLAN-7 antibodies in oxalate, carbonate, and chlorite extracts (Figs. 3, 4) suggesting enhanced reactivity of this enzyme with homoxylan in comparison with the other two. In general, there was an overall subtle decrease in the binding of XYLAN-4 (detecting arabinosylated xylan epitopes) and XYLAN-5 (methyl GlcA-substituted xylans) mAbs groups, especially against oxalate and carbonate extracts from XynY treated biomass residues of CS and SG. This suggests that XynY is potentially more active than the other two enzymes in the hydrolysis of highly substituted regions of xylans. XynY was able to remove the 1 M KOH extractable xyloglucan epitopes from AFEX-treated CS (similar to CMX00_3a), but did not fully remove these epitopes in AFEX-treated SG.

The mAb screening of supernatants (Fig. 5) supported the conclusions drawn from the glycome profiling of the residual solids after treatment with XynY. No epitopes recognized by the XYLAN-7 antibodies were detected in the supernatants, and the extent of binding of the XYLAN-5 antibodies was reduced in comparison with the results obtained with either CMX00_3a or XynA.

### Effects of XynA activity

The glycome profiles of AFEX pretreated CS and SG residues treated with XynA (GH11) demonstrated that XynA had the narrowest substrate specificity among the three enzymes examined. Unlike the other two enzymes, XynA was not effective in removing any significant amounts of the xylan epitopes that are recognized by the XYLAN-3, -4 or -5 groups of mAbs. Further, the overall data show that XynA is not as effective as either CMX00_3a or XynY in removing the xylan epitopes detected by the XYLAN-7 antibodies (CCRC-M152 and CCRC-M149, Figs. 3, 4, 5). XynA also appeared to have limited capability to remove xyloglucan epitopes from either AFEX-treated CS or SG.

### Oxime-NIMS studies

Oxime-NIMS allows quantitative detection of soluble reducing sugars released from enzyme hydrolysis of plant biomass [48]. In grasses, glucose is the dominant hexose reducing sugar, while xylose is the dominant pentose reducing sugar (see “Methods” section, enzyme reactions for the composition of corn stover and switchgrass used in this work). In the following, we distinguish between oligosaccharides containing hexose reducing sugars only (i.e., glucose, cellobiose, cellotriose, cellotetraose derived primarily from cellulose), others containing pentose reducing sugars only, and some additional oligosaccharides that contain a mixture of hexose and pentose sugars. Oxime-NIMS cannot distinguish between stereoisomers with the same mass, such as glucose and galactose, or xylose and arabinose.

Oxime-NIMS analysis of soluble products from enzyme reactions with both AFEX-CS and AFEX-SG (Tables 3, 4) showed that negligible amounts of reducing sugar were present in the supernatants either with or without AFEX treatment, while a greater than 50× increase in sugar release was observed by the addition of any of the three enzymes to the AFEX-treated material.

**Table 3:** Enzyme activities for AFEX-CS hydrolysis measured by oxime-NIMS

	G1 (mM)	G2 (mM)	G3 (mM)	G4 (mM)	Total (mM)	Average (mM)	Stdev		
No enzyme	0.3	0.0	0.0	0.0	0.3	–	–		
CMX00_3a	4.7	5.6	0.1	0.0	10.4				
	4.9	8.2	0.2	0.1	13.4	11.9	2.1		
XynY	0.0	0.0	1.2	0.2	1.5				
	0.0	0.0	1.4	0.3	1.7	1.6	0.2		
XynA	0.0	0.0	0.1	0.0	0.1				
	0.0	0.0	0.1	0.0	0.1	0.1	0.0		

	P1 (mM)	P2 (mM)	P3 (mM)	P4 (mM)	P5 (mM)	P6 (mM)	Total (mM)	Average (mM)	Stdev
No enzyme	0.0	0.0	0.0	0.0	0.0	0.0	0.0	–	–
CMX00_3a	2.2	10.4	13.1	4.4	1.2	0.2	31.5		
	2.0	13.2	19.2	7.7	2.8	0.6	45.5	38.5	9.9
XynY	95.2	129.1	27.5	9.5	1.1	0.1	262.4		
	84.7	138.3	30.2	13.8	1.7	0.1	268.8	265.6	4.5
XynA	31.8	73.3	0.2	2.1	10.5	1.0	118.8		
	38.2	78.3	0.2	2.1	8.0	0.7	127.4	123.1	6.1

	X^a^ (mM)	XG (mM)	X2-G (mM)	X3-G (mM)	Xyl-Rha-GalA-Xyl (mM)	Total (mM)	Average (mM)	Stdev	
No enzyme	0.0	0.0	0.0	0.0	0.0	0.0	–	–	
CMX00_3a	2.8	0.1	1.1	0.2	1.5	5.7			
	4.0	0.2	2.2	0.4	2.3	9.1	7.4	2.4	
XynY	0.7	0.0	0.1	0.1	7.7	8.6			
	0.6	0.0	0.2	0.1	10.1	11.0	9.8	1.7	
XynA	0.4	0.0	0.0	0.0	0.4	0.9			
	0.4	0.0	0.0	0.0	0.5	0.9	0.9	0.0	

Activity reported as µmol of reducing sugar released per hour per µmol of purified enzyme determined by oxime-NIMS as described in “Methods” section. Standard deviation indicated for the duplicate samples

*AFEX-CS* AFEX-treated corn stover, *G1* glucose, *G2* cellobiose, *G3* cellotriose, *G4* cellotetraose, *P1* pentose (i.e., likely xylose), *P2* pentobiose, *P3* pentotriose, *P4* pentotetraose, *P5* pentopentose, *P6* pentohexose

^a^Xyloglucan-derived oligosaccharides identified using abbreviations from [59]; X, Xyl–Glu; XG, Xyl–Glu–Glu; X2–G, Xyl–Xyl–Glc, X3–G, Xyl–Xyl–Xyl–Glc

**Table 4:** Enzyme activities for AFEX-SG hydrolysis measured by oxime-NIMS

	G1 (mM)	G2 (mM)	G3 (mM)	G4 (mM)	Total (mM)	Average (mM)	Stdev		
No enzyme	0.2	0.0	0.0	0.0	0.58	–	–		
CMX00_3a	4.6	5.8	0.2	0.1	8.54				
	4.5	5.0	0.1	0.0	7.67	10.2	0.8		
XynY	0.0	1.3	1.5	0.2	0.76				
	0.0	1.0	1.0	0.2	0.54	2.6	0.6		
XynA	0.0	0.0	0.0	0.0	0.02				
	0.0	0.0	0.0	0.0	0.00	0.0	0.0		

	P1 (mM)	P2 (mM)	P3 (mM)	P4 (mM)	P5 (mM)	P6 (mM)	Total (mM)	Average (mM)	Stdev
No enzyme	0.0	0.0	0.0	0.0	0.0	0.0	0.0	–	–
CMX00_3a	3.2	14.5	17.3	6.7	1.6	0.3	43.6		
	3.1	15.3	17.0	6.6	1.1	0.2	43.3	43.4	0.2
XynY	94.5	127.9	40.5	10.5	1.1	0.1	274.6		
	93.1	143.3	40.1	12.0	0.9	0.0	289.5	282.0	10.5
XynA	36.7	94.5	0.3	2.2	10.7	1.0	145.3		
	39.3	95.0	0.1	1.5	11.0	0.3	147.3	146.3	1.4

	X^a^ (mM)	XG (mM)	X2-G (mM)	X3-G (mM)	Xyl-Rha-GalA-Xyl (mM)	Total (mM)	Average (mM)	Stdev	
No enzyme	0.0	0.0	0.0	0.0	0.0	0.0	–	–	
CMX00_3a	2.9	0.1	1.4	0.2	0.6	5.2			
	1.4	0.1	1.1	0.2	0.6	3.4	4.3	1.3	
XynY	0.4	0.0	0.2	0.1	2.6	3.3			
	0.4	0.0	0.2	0.1	2.5	3.1	3.2	0.1	
XynA	0.4	0.0	0.0	0.0	0.1	0.5			
	0.2	0.0	0.0	0.0	0.1	0.3	0.4	0.1	

Activity reported as µmol of reducing sugar released per hour per µmol of purified enzyme determined by oxime-NIMS as described in “Methods” section. Standard deviation indicated for the duplicate samples

*AFEX-SG* AFEX-treated switchgrass, *G1* glucose, *G2* cellobiose, *G3* cellotriose, *G4* cellotetraose, *P1* pentose (i.e., likely xylose), *P2* pentobiose, *P3* pentotriose, *P4* pentotetraose, *P5* pentopentose, *P6* pentohexose

^a^Xyloglucan-derived oligosaccharides identified using abbreviations from [59]; X, Xyl–Glu; XG, Xyl–Glu–Glu; X2–G, Xyl–Xyl–Glc, X3–G, Xyl–Xyl–Xyl–Glc

**Fig. 6:**
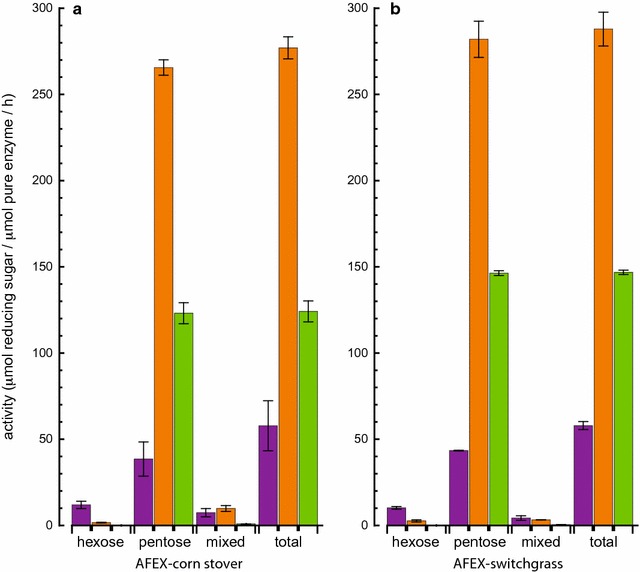
Oxime-NIMS analysis of enzyme activity (µmol of reducing sugar released per µmol of pure enzyme per hour at 55 °C) for different classes of reducing sugar products obtained from enzymatic hydrolysis of AFEX-corn stover or AFEX-switchgrass. Hexose products are derived from cellulose, while pentose and mixed pentose/hexose products (e.g., for mixed products, Xyl–Glc, Xyl–Xyl-Glc, Glc–Glc–Xyl, Xyl–Xyl–Xyl–Glc, Xyl–Rha–GalA–Xyl) are from various hemicellulose polysaccharides. Tables 3 and 4 list the concentrations of individual soluble oligosaccharides detected by NIMS in each of these categories. The *color bars* depict the different enzymes used: CMX00_3a (*purple*); XynY (*orange*); XynA (*green*)

Figure 6 and Tables 3, 4 show that CMX00_3a released cello-oligosaccharides from the AFEX-treated biomass. At the endpoint of the reaction, CMX00_3a produced mostly cellobiose and glucose and lesser amounts of cellotriose and cellotetraose. These products are in accord with previous studies of the CMX00_3a reaction with IL-treated switchgrass, although a considerably higher yield of cellulose-derived products was observed from the IL-treated biomass than the AFEX-treated biomass [48]. XynY and XynA released pentose-containing oligosaccharides, consisting primarily of xylose and xylobiose (Tables 3, 4), which is in accord with their activities on pure xylan (Table 2). CMX00_3a produced approximately half of the total pentose-derived products given by XynY and XynA (Tables 3, 4), but with a broader distribution of products and with xylotriose as the dominant product, as was observed previously with IL-treated SG [48].

Oxime-NIMS also revealed the formation of mixed pentose-hexose products, which are likely derived from substructures within xyloglucans or xylans (Fig. 6; Tables 3, 4). CMX00_3a yielded these as ~8% of its total hydrolysis products from both corn stover and switchgrass, while reactions with XynA and XynY released 3% or less of these mixed products. The masses of the mixed products are consistent with the following assignments, using the nomenclature for abbreviating xyloglucan substructures developed earlier [68]: xylose-1-α-6-glucose (abbreviated X, or less likely galactose-1-β-2-xylose); and (xylose-1-α-6-glucose)-1-β-4-glucose (XG). XG was dominant product observed from all enzymes (Tables 3, 4).

In addition, we observed mixed products with masses consistent with hexose-(pentose)_2_, and hexose-(pentose)_3_, which are likely derived from substructures of xylan (Fig. 6; Tables 3, 4). CMX00_3a and XynY had similar activity for release of an oxime-NIMS product with *m/z* = 1379.56 (~4% total solubilized sugar), which is consistent with an assignment to Xyl–Rha–GalA–Xyl (621.56 Da), a tetrasaccharide previously identified as being present at the reducing end of some xylans [69]. This tetrasaccharide, which was detected in a larger amount in these samples of corn stover, has not been previously identified in grasses [70–72], but has been identified in dicots [73] and woody plants [74, 75].

## Discussion

Individual enzymes from three different GH families known to hydrolyze hemicellulosic polysaccharides were studied for their activities on representative purified forms of hemicelluloses (e.g., oat spelt xylan, tamarind seed xyloglucan, wheat arabinoxylan) and also for their reactions with AFEX-treated corn stover and switchgrass using glycome profiling [45] and quantitative oxime-NIMS [48]. The results presented here demonstrate that the combination of glycome profiling and oxime-NIMS allows one to clearly distinguish the three activities exhibited by these enzymes in reactions with plant biomass. Extension of this approach to other enzymes, different polysaccharides, and different plant biomasses has the potential to extend our specificity of understanding of enzyme specificity in a biofuel context. For example, pectic polysaccharides provide challenging, diverse structures that could be studied by the approach reported here. These studies would be enhanced by the use of additional techniques sensitive to stereochemical differences in sugars, such as high performance liquid or gas chromatography [76–79], which potentially allow further insight into the pentose product cascades identified by oxime-NIMS.

Table 5 provides a summary of the breadth of substrates hydrolyzed by each individual enzyme. The epitope recognized by XYLAN-6 antibodies consists of at least four consecutive, unsubstituted β-1,4 linked xylose units [80] (also see Additional file 1: Table S1), and hydrolysis at any position within this epitope will lead to a loss of antibody binding. All four antibodies in the XYLAN-7 group bind to linear xylans of DP 4 or higher and can tolerate at least some level of arabinosyl substitution along the backbone [80]. XYLAN-7 antibodies recognize epitopes that consist of at least three β-1,4 linked xylose units; these antibodies can tolerate varying levels of arabinosyl substitution of the backbones [80]. CCRC-M154 binds to the arabinosyl side-chains of xylans; the antibody does not bind to the backbone xylosyl residues at all [80]. The XYLAN-5 antibodies bind to 4-*O*-methyl glucuronic acid substituted xylans that carry no acetyl substituents (unpublished results of the Hahn lab, in preparation).

**Table 5:** Summary of enzyme specificities detected by glycome profiling or oxime-NIMS

Enzyme	GH family	Substrates identified^a^
CMX00_3a	GH5	Oat spelt xylan (β-1,4-xylose)^b^
		Tamarind seed xyloglucan^c^
		Cellulose (β-1,4-glucose)
		NON-FUC XG epitopes (structures not known)
		FUC-XG epitopes (structures not known)
		XYLAN-3 epitopes (structures not known)
		XYLAN-6 epitopes (unsubstituted xylan)^d^
		XYLAN-7 epitopes (unsubstituted xylan)^e^
		CCRC-M160
		CCRC-M137
XynY	GH10	Oat spelt xylan (β-1,4-xylose)^b^
		Wheat arabinoxylan^f^
		XYLAN-3 epitopes, structures not known
		XYLAN-4 epitopes
		CCRC-M154, arabinosylated-xylan
		XYLAN-5 epitopes, methyl GlcA-substituted xylan
		XYLAN-6 epitopes (unsubstituted xylan)^4^
		XYLAN-7 epitopes (unsubstituted xylan)^5^
		CCRC-M160
		CCRC-M137
		CCRC-M152
		CCRC-M149
XynA	GH11	Oat spelt xylan (β-1,4-xylose)^b^
		Tamarind seed xyloglucan^c^
		Wheat arabinoxylan^f^
		XYLAN-6 epitopes (unsubstituted xylan)^d^
		XYLAN-7 epitopes (unsubstituted xylan)^e^
		CCRC-M160
		CCRC-M137

^a^Reactions with purified hemicellulose polysaccharides detected by DNS; reaction with cellulose in AFEX-treated CS and SG detected by oxime-NIMS; reaction of hemicellulose fraction in CS and SG detected by glycome profiling and oxime-NIMS

^b^β-1,4-xylose with ~70:15:10 composition of xylose:glucose:arabinose

^c^β-1,4-glucose with α-1,6 xylose branching at on average, 3 out of 4 glucosyl backbone residues

^d^Stretches of unsubstituted β-1,4-xylan with at least four xylose units

^e^Stretches of unsubstituted β-1,4-xylan with at least three xylose units

^f^β-1,4-xylose with 38:62 composition of arabinose and xylose, less than 1% of glucose, galactose, and mannose

XynY (GH10 xylanase) and XynA (GH11 xylanase) showed the highest specific activities with the oat spelt xylan polysaccharides and with the AFEX-treated biomass, consistent with their previously determined hydrolytic capabilities [48, 53, 54]. The presence of CBM6 in XynA and CBM22 in XynY, which both bind xylan, enhances their reactivity in ways that have been observed for other enzyme-CBM fusions [50, 81, 82]. In contrast, CBM3a present in CMX00_3a does not bind well to xylan [59, 60, 63], which may have contributed to the lower specific activity for hydrolysis of xylan substrates. The ability of fusions of CBMs and catalytic domains to alter enzyme activity is widely known [48, 50, 81, 83–89]. Along this line, in our other work with CMX00, we showed that fusion to CBM44, which shows preferential binding to xylan relative to cellulose, yielded an enzyme that was more reactive with xylan and the hemicellulose fraction of IL-pretreated SG [50].

The activities of XynY and XynA could be clearly distinguished from one another, and from the activity of CMX00_3a. XynY appears to have the broadest activity against xylans in the plant cell wall, and completely or nearly completely degraded the epitopes recognized by the XYLAN-6 and XYLAN-7 antibodies, particularly in the oxalate, carbonate and chlorite extracts, and also cleaved essentially all epitopes bound by the XYLAN-3 antibodies in all wall extracts. XynY digestion of biomass also resulted in degradation of epitopes recognized by the XYLAN-4 and XYLAN-5 antibodies, again most prominently in the oxalate and carbonate extracts as well as in the supernatants from the enzyme reactions. XynY was also the most active enzyme on the glucuronoarabinoxylan epitopes, as judged by decrease in the intensity of XYLAN-4 and XYLAN-5 in the glycome profiles and its reaction with arabinoxylan (Table 1; Fig. 2). This broad specificity is reasonable because many other members of GH10 are known to hydrolyze glycosidic bonds regardless of whether branched or unbranched xylose units are placed at the +1 and −1 positions of the catalytic site. In contrast, CMX00_3a (GH5) and XynA (GH11) are from GH families where the +1 and −1 sites of the catalytic sites are typically less accommodating of branching substitutions [55, 56]. Tolerance of substitutions at the +1 and −1 positions would likely permit more options for reaction along a typical arabinoxylan chain, where up to 40% of the backbone molecules contain a branching substituent.

CMX00_3a was able to hydrolyze cellulose present in the pretreated biomass, which was not detected by glycome profiling, but was confirmed by oxime-NIMS (Tables 3, 4). The oxime-NIMS analysis also showed that neither XynY nor XynA hydrolyzed cellulose to any appreciable extent. CMX00_3a also reacted with xylan epitopes detected by the XYLAN-3, XYLAN-6, and XYLAN-7 antibodies, particularly in the oxalate and carbonate extracts, and also with epitopes recognized by antibodies for xyloglucan structures (Table 5).

XynA was the most selective of the three enzymes studies here. This enzyme was only effective in hydrolyzing epitopes recognized by the XYLAN-6 antibodies in the oxalate and carbonate wall extracts and in the enzyme supernatants. XynA also hydrolyzed epitopes recognized by the XYLAN-7 antibodies, CCRC-M137 and CCRC-M160, while apparently not hydrolyzing xylans recognized by the other two XYAN-7 antibodies, CCRC-M149 and CCRC-M152. XynA was ineffective in degrading epitopes recognized by the XYLAN-3, -4 and -5 antibodies, nor was it able to effectively catalyze removal of xyloglucan epitopes.

The three enzymes had markedly different reactivities with isolated tamarind xyloglucan. As detected by DNS, CMX00_3a and XynA were able to degrade tamarind xyloglucan, while XynY showed only ~1/1000 of their activities (Fig. 2). Thus, it appears that XynY is not able to cleave the unbranched glucosyl residues in the xyloglucan backbone, while CMX00_3a and XynA are able to cleave these residues to a certain extent, at least in isolated tamarind xyloglucan. Interestingly, XynA appeared to have no effect on xyloglucans in AFEX pre-treated biomass samples. The reactivity of CMX00_3a (GH5_4) with tamarind seed xyloglucan as detected by DNS and MALDI-MS was consistent with its ability to degrade xyloglucan epitopes in the 1 M KOH extracts of pretreated grasses as judged by glycome profiling (Figs. 4, 5) and by the appearance of mixed pentose-hexose products detected by oxime-NIMS. While oxime-NIMS did not detect small soluble products of tamarind seed xyloglucan hydrolysis (e.g., glucose, xylose), MALDI showed accumulation of longer insoluble xyloglucan oligosaccharides such as Glc_4_-Xyl_3_, Glc_5_-Xyl_4_, and Glc_6_-Xyl_5_, representing highly branched portions of the polysaccharide that could not be hydrolyzed.

Xylanases from GH11 have not been previously reported to hydrolyze β-1,4 linked glucose [57, 58, 90], which forms the backbone of xyloglucan. However, xylanases from GH11 and cellulases from GH12 share the same β-jelly roll protein fold and have spatially comparable active site residues. Thus, XynA may have other active site feature(s) of GH12 needed for reaction with substituted β-1,4 linked glucose backbones [58, 91, 92]. This potential broadening of specificity in the β-jelly roll fold of GH11 and GH12 can be contrasted with the specificities of the (αβ)_8_ barrel folds of CMX00 (GH5_4) and (XynY) GH10, where CMX00 reacts with cellulose (β-1,4 linked glucose), xylan (β-1,4 linked xylose), and xyloglucan (β-1,4 linked glucose with α-1,6 linked branching), while XynY only reacted with xylan (β-1,4 linked xylose) and arabinoxylan and did not react with either xyloglucan or cellulose [54, 58, 90, 93]. Identification of the features that control divergent specificity for polysaccharide backbones and tolerance to branching in closely related protein folds is a matter of ongoing investigation in many laboratories.

## Conclusions

Glycome profiling was used to observe the consequences of enzyme hydrolysis on AFEX-treated plant cell walls and proved capable of distinguishing the activities of related enzymes. Enzymes from GH5, GH10, and GH11 were demonstrated to hydrolyze at least some xyloglucan and/or xylans both in vitro and in AFEX-pretreated biomass. The GH10 enzyme, XynY, proved to be reactive with the broadest diversity of xylose-backbone polysaccharide epitopes, but was incapable of reacting with glucose-backbone polysaccharides. In contrast, the GH5 and GH11 enzymes studied here showed the ability to react with both glucose- and xylose-backbone polysaccharides (at least in vitro), a potentially useful breadth of specificity given the complexity of plant biomass. The methods used in this work provide a complementary view of GH function with relatively intact plant cell walls, and offer new insights into how additional specific hemicellulose substructures could be targeted for more efficient hydrolysis by simple combinations of pretreatment, enzymes, and CBMs.

## Methods

### Cloning, expression, and purification of enzymes

The cloning of CMX00_3a and XynY into the wheat germ cell-free translation plasmid pEU was described previously [48, 52]. The cloned gene sequences were transferred from the cell-free expression plasmid by digestion with *Sgf*I and *Bam*HI, and ligation into similarly digested pVP67K, a lactose inducible expression vector for use in *Escherichia coli* [94].

For protein expression, pVP67K plasmids were transformed into competent *Escherichia coli* BL21-CodonPlus (DE3)-RILP cells (Agilent Technologies, Santa Clara, CA, USA). CMX00_3a and XynY used in this work were produced using auto-induction expression [95] at the DOE Advanced Bioprocessing Demonstration Unit (Lawrence Berkeley National Laboratory, Emeryville, CA, USA) and purified using HisTrap column chromatography at KanPro Research, Inc. (Lawrence, KS, USA). The yield of purified CMX00_3a after expression in *E. coli* and purification using metal affinity chromatography was ~0.45 g/L of culture medium. The yield of purified XynY after expression in *E. coli* and Ni^2+^-immobilized metal affinity chromatography was ~0.6 g/L of culture medium.

XynA was cloned from *R. thermocellum* genomic DNA using forward primer 5′-CTGTACTTCCAGGCGATCGCCgatgtagtaattacgtcaaaccagacg-3′ and reverse primer 5′-TCGAATTCGTTTAAACTACTAcgagtcgaatatgaagtagtcaatgtt-3′, and polymerase incomplete primer extension [96]. The plasmid pVP67K was amplified with forward primer 5′-GGCGATCGCCTGGAAGTACAG-3′ and reverse primer 5′-TAGTAGTTTAAACGAATTCGA-3′. Uppercase letters represent the overlapping nucleotides of the amplified XynA gene and pVP67K that are annealed during transformation, and the lowercase letters are the nucleotides of the XynA gene. The cloned XynA consisted of the GH11 catalytic domain and a C-terminal CBM6 domain. pVP67K and XynA were amplified in separate PCR reactions, then 2 μL of each were mixed together and transformed into competent *E. cloni* 10G cells (Lucigen Corporation, Middleton, WI, USA). Kanamycin-resistant transformants were sequenced to identify inserts with the correct gene sequence using forward primer 5′-TTGCTTTGTGAGCGGATAAC-3′ and reverse primer 5′-GCTAGTTATTGCTCAGCGG-3′.

XynA was expressed using auto-induction and purified as follows: Cells containing the XynA plasmid were grown for 12 h at room temperature in 500-mL Erlenmeyer flasks containing 50 mL of non-inducing medium and subsequently split and transferred into two 2-L plastic baffled bottles containing 0.5 L of auto-induction medium for 25 h with shaking at room temperature [95]. The cells were harvested by centrifugation; the cell pellet was re-suspended in 100 mM MOPS, pH 7.4, containing 0.5 M NaCl, 20 mM imidazole, 1 mM EDTA, 1.0 μM E-64 (Sigma-Aldrich, St. Louis, MO, USA), and 0.5 mM benzamidine (EMD Millipore, Darmstadt, Germany). The suspended cells were sonicated with a 15-s on/off cycle on wet ice for a total of 10-min sonication time. The sonicated lysate was centrifuged and the supernatant was loaded onto a HisTrap column (GE Healthcare, Piscataway, NJ, USA) equilibrated with 100 mM MOPS, pH 7.4, containing 0.5 M NaCl and 20 mM imidazole. After lysate loading, the column was washed with ~3 column volumes of the equilibration buffer and then a linear gradient with 100 mM MOPS, pH 7.4, containing 0.5 M NaCl, and 0.5 M imidazole was used to elute the protein. Fractions containing pure protein were identified by denaturing gel electrophoresis, pooled, concentrated, and buffer exchanged into 10 mM MOPS, pH 7 containing 50 mM NaCl using Vivaspin 20 centrifugal concentrators (Sartorius Stedim, Bohemia, NY, USA). The yield of purified XynA after expression in *E. coli* and Ni^2+^-immobilized metal affinity chromatography was ~0.3 g/L of culture medium.

### Enzyme reactions

Untreated and ammonia fiber expansion (AFEX) pretreated corn stover and switchgrass [33] grown during 2010 were provided by Dr. Bruce E. Dale (Great Lakes Bioenergy Research Center). Compositional analyses of the corn stover and switchgrass were performed using NREL procedures LAP-002 and LAP-005 [97–99]. For corn stover, the percentage compositions of the untreated, dry biomass (w/w) were 30.7% glucan, 18.5% xylan, 1.2% galactan, 3.1% arabinan, 14.3% lignin, 3.2% protein, and 13.4% ash. For switchgrass, the percentage compositions of the untreated, dry biomass was 34.2% (w/w) glucan, 21.5% xylan, 1.4% galactan, 2.3% arabinan, 18.6% lignin, 3.2% protein, and 5.6% ash. Oat spelt xylan (>70% purity with ~70:15:10 composition of xylose:glucose:arabinose) was from Sigma-Aldrich (St. Louis, MO, USA), and tamarind xyloglucan (~95% purity with 3:18:34:45 composition of arabinose:galactose:xylose:glucose) and low viscosity wheat arabinoxylan (~95% purity with 38:62 composition of arabinose and xylose, less than 1% of glucose, galactose, and mannose) were from Megazyme (Wicklow, Ireland).

Oat spelt xylan was prepared [100, 101] by boiling two grams of the polysaccharide in 100 mL of distilled water for 30 min and subsequently pelleting the insoluble fraction by centrifugation for 20 min at 4300×*g* at 4 °C. The insoluble xylan pellet was washed three times by centrifugation for 20 min at 4300×*g* at 4 °C and placed at −80 °C overnight. The sample was lyophilized to obtain ~1 g of the final insoluble substrate.

Enzyme assays with pure substrates contained 1 mg of either xylan, xyloglucan, or arabinoxylan mixed with 20 μg of CMX00_3a (60,118 Da, 0.33 nmol of active sites), 8 μg of XynY (78,573 Da, 0.10 nmol of active sites), or 8 μg of XynA (40,317 Da, 0.20 nmol of active sites) in 100 μL of 50 mM sodium phosphate, pH 6, containing 1 mg/mL bovine serum albumin to mitigate non-specific binding of enzyme. Control reactions with no enzyme substituted buffer for the volume of added enzyme. Reactions were shaken at 1200 rpm and 55 °C within a Heidolph Titramax 1000/Incubator 1000 combination (Heidolph North American, Elk Grove Village, IL, USA). After 24 h, the reactions were cooled on ice for 5 min to stop the reactions and centrifuged at 4000×*g* for 10 min at 4 °C, and the supernatant fractions were collected for the determination of reducing sugar amounts using the dinitrosalicylic acid (DNS) assay [50, 52, 102]. For the DNS reaction, the appropriately diluted supernatant (30 µL) was added to 60 μL of DNS reagent [1% (w/v) 3,5-dinitrosalicylic acid, Sigma-Aldrich, 0.2% (v/v) phenol, 0.05% (w/v) sodium sulfite, and 1% (w/v) sodium hydroxide] and boiled at 95 °C for 5 min. After boiling, the absorbance of the samples was measured at 540 nm, and d-glucose standards were used to calculate reducing sugar concentrations. All reactions were performed in triplicate.

For enzyme reactions to prepare solid samples for glycome profiling and supernatant samples for antibody screening and for oxime-NIMS, 1 g of substrate (i.e., untreated or AFEX-treated corn stover or switchgrass) was mixed with 20 mg of CMX00_3a (0.3 µmol of active sites), 8 mg of XynY (0.1 µmol of active sites), or 8 mg of XynA (0.2 µmol of active sites) in 10 mL of 50 mM sodium phosphate, pH 6, containing 1 mg/mL bovine serum albumin. All reactions were performed in duplicate. In control samples lacking enzyme, 10 mM MOPS, pH 7, containing 50 mM NaCl was used to replace the volume of enzyme added. Reactions were incubated at 55 °C while shaking on a Thermo Scientific Titer Plate Shaker (Model No. 4625) (Thermo Fisher Scientific, Waltham, MA, USA) for 24 h. Indicated control reactions and all enzyme reactions were stopped by placing the reaction vessel in a 95 °C water bath for 5 min (‘boiled’ samples). The samples were centrifuged at 4000×*g* for 15 min at 4 °C, and the supernatant and solid fractions were separated. The supernatant fractions were filtered using a 0.2 μm filter and frozen with liquid N_2_. Both supernatant and solid fractions were stored at −80 °C until needed. All supernatants and cell wall extracts were lyophilized before glycome profiling processing. Aliquots (100 µL) of the filtered supernatants were taken to measure reducing sugar concentrations using the DNS assay and to perform oxime-NIMS analyses [48, 49]. The DNS assay was carried out as mentioned above. Oxime-NIMS methods are described below.

### Glycome profiling

Glycome profiling involves using an increasingly harsh series of extractions of plant cell wall material to yield wall extracts that contain mixtures of most major non-cellulosic cell wall glycans including hemicelluloses and pectic polysaccharides [45, 47]. These extracts are then screened by ELISA [45] using a comprehensive suite of cell wall glycan-directed mAbs [44, 45, 103, 104] for detecting diverse glycan epitopes present in the non-cellulosic glycans (Additional file 1: Table S1). Loosely attached polysaccharides are removed during the early extractions (oxalate and carbonate) while more tightly bound polysaccharides are freed by the harshest extractions (1 M KOH, 4 M KOH, chlorite, and 4 M KOHPC).

Glycome profiling of various biomass samples (including untreated, pretreated and enzyme hydrolyzed samples) was done as described earlier [37, 45, 46, 71]. Supernatants were also subjected to screening with the entire collection of cell wall glycan-directed monoclonal antibodies used for glycome profiling. The cell wall glycan-directed antibodies used in glycome profiling analyses and screening of supernatants [44] were procured from laboratory stocks (CCRC, JIM, and MAC series) at the Complex Carbohydrate Research Center (available through CarboSource Services; http://www.carbosource.net) and also from BioSupplies (Australia) (BG1, LAMP).

### Oxime-NIMS methods

Soluble products in the supernatants of the hydrolysis reactions of various biomass samples (including untreated, pretreated and enzyme hydrolyzed samples) were detected using oxime-NIMS. Synthesis of the *O*-alkyloxyamine fluorous tagged NIMS reagent has been published [48]. Supernatant samples for oxime-NIMS analysis were prepared as described above. A 2 µL aliquot of the supernatant was transferred into a vial containing 6 µL of 100 mM glycine acetate, pH 1.2, 1.0 µL of a mixture of 2.5 mM aqueous solution of [*U*]-^13^C glucose and 2.5 mM aqueous solution of [*U*]-^13^C xylose, 2 µL of CH_3_CN, 1 µL of MeOH, 1 µL of the NIMS probe (100 mM in 1:1 (v/v) H_2_O:MeOH), and 0.1 µL of aniline. The sample was incubated at room temperature for 16 h, and then a 0.12 µL aliquot was spotted onto the surface of the NIMS chip and removed after 30 s. A grid drawn manually on the NIMS chip using a diamond-tip scribe helped with spotting and identification of sample spots in the spectrometer. NIMS chips were loaded using a modified standard MALDI plate and analyzed using a 5800 MALDI TOF/TOF mass spectrometer (Applied Biosystems, Foster City, CA, USA). Signal intensities were identified for product ions and ~1000 laser shots were collected for each sample spot. Product quantitation was achieved by using either [*U*]-^13^C glucose or [*U*]-^13^C xylose as an internal standard.

### MALDI-MS methods

High molecular weight insoluble products in the supernatants of the hydrolysis reactions of tamarind seed xyloglucan hydrolysis were detected using MALDI-MS. The matrix used was either Universal MALDI Matrix (Aldrich, St. Louis, MO, USA) or α-cyano-4-hydroxy-cinnamic acid (Aldrich, St. Louis, MO, USA) dissolved in methanol with 0.1% trifluoroacetic acid with concentration of 30 mg/mL. The samples and the matrices were mixed in a 1:1 ratio and 0.5 μL of the resulting mixture were deposited onto an Opti-TOF 384 well MALDI plate. The air-dried samples were analyzed using a 5800 MALDI TOF/TOF mass spectrometer (Applied Biosystems, Foster City, CA, USA). The operating mode was either MS Reflector Positive or Linear Mid Mass Range.

## Additional file

**Additional file 1: Table S1.** Detailed list of cell wall glycan-directed monoclonal antibodies (mAbs) used for glycome profiling analyses.

## Abbreviations

GH: glycoside hydrolase
CBM: carbohydrate binding module
CMX00: broad specificity GH family 5 (GH5) domain from *R. thermocellum* Cthe_0797
AFEX-CS: ammonia fiber expansion-pretreated corn stover
AFEX-SG: ammonia fiber expansion-pretreated switchgrass
NIMS: nanostructure-initiator mass spectrometry
