# Supplementary material for: Determination of glycoside hydrolase specificities during hydrolysis of plant cell walls using glycome profiling
Source: Biotechnol Biofuels. 2017 Feb 2;10:31. doi: 10.1186/s13068-017-0703-6 (PMC5288845; doi:10.1186/s13068-017-0703-6)
Supplement: Supplementary file 1 — Additional file 1: Table S1. Detailed list of cell wall glycan-directed monoclonal antibodies (mAbs) used for glycome profiling analyses. [file 13068_2017_703_MOESM1_ESM.pdf]

Supporting Information Table S1

Detailed list of cell wall glycan-directed monoclonal antibodies (mAbs) used for glycome profiling analyses. The groups of antibodies are based on a hierarchical clustering of ELISA data generated from a screen of all mAbs against a comprehensive panel of plant polysaccharide preparations [1, 2] that clusters mAbs according to the predominant polysaccharides recognized. The majority of listings link to the Wall*Mab*DB plant cell wall monoclonal antibody database (http://www.wallmabdb.net) that provides detailed descriptions of each mAb, including immunogen, antibody isotype, epitope structure (to the extent known), supplier information, and related literature citations.

Glycan Group Recognized	mAb Names	Epitope (If Known)
Non-Fucosylated Xyloglucan-1	CCRC-M95	
	CCRC-M101	
Non-Fucosylated Xyloglucan-2	CCRC-M104	
	CCRC-M89	
	CCRC-M93	
	CCRC-M87	
	CCRC-M88	
Non-Fucosylated Xyloglucan-3	CCRC-M100	
	CCRC-M103	
Non-Fucosylated Xyloglucan-4	CCRC-M58	
	CCRC-M86	
	CCRC-M55	
	CCRC-M52	
	CCRC-M99	
Non-Fucosylated Xyloglucan-5	CCRC-M54	
	CCRC-M48	
	CCRC-M49	
	CCRC-M96	
	CCRC-M50	
	CCRC-M51	
	CCRC-M53	

Non-Fucosylated Xyloglucan-6	CCRC-M57	
Fucosylated Xyloglucan	CCRC-M102	
	CCRC-M39	
	CCRC-M106	
	CCRC-M84	
	CCRC-M1	(α-Fuc-(1,2)-β-Gal) epitope in fucosylated xyloglucan [3]
Xylan-1/XG	CCRC-M111	
	CCRC-M108	
	CCRC-M109	
Xylan-2	CCRC-M119	
	CCRC-M115	
	CCRC-M110	
	CCRC-M105	
Xylan-3	CCRC-M117	
	CCRC-M113	
	CCRC-M120	
	CCRC-M118	
	CCRC-M116	
	CCRC-M114	
Xylan-4	CCRC-M154	Arabinosylated xylan epitopes [4]
	CCRC-M150	Both homo- and arabinosylated xylan epitopes [4]
Xylan-5	CCRC-M144	
	CCRC-M146	
	CCRC-M145	Methyl GlcA substituted xylan (Hahn, M.G, unpublished data)

	CCRC-M155	Methyl GlcA substituted xylan (Hahn, M.G, unpublished data)
Xylan-6	CCRC-M153	Homoxylan epitopes DP>4 [4]
	CCRC-M151	Homoxylan epitopes DP>4 [4]
	CCRC-M148	Homoxylan epitopes DP>4 [4]
	CCRC-M140	Homoxylan epitopes DP>4 [4]
	CCRC-M139	Homoxylan epitopes DP>4 [4]
	CCRC-M138	Homoxylan epitopes DP>4 [4]
Xylan-7	CCRC-M160	Homoxylan epitopes DP>3 [4]
	CCRC-M137	Homoxylan epitopes DP>3 [4]
	CCRC-M152	Homoxylan epitopes DP>3 [4]
	CCRC-M149	Homoxylan epitopes DP>3 [4]
Galactomannan-1	CCRC-M75	
	CCRC-M70	
	CCRC-M74	
Galactomannan-2	CCRC-M166	
	CCRC-M168	
	CCRC-M174	
	CCRC-M175	
Acetylated Glucomannan	CCRC-M169	[5]
	CCRC-M170	
β-Glucan	LAMP	1,3 β-glucan [6]
	BG1	1,3; 1,4 β-glucan [7]

HG Backbone-1	CCRC-M131	
	CCRC-M38	de-esterified α-1,4 linked homogalcturonan (HG) epitope [1]
	JIM5	Partially methyl esterified or de-esterified α-1,4 linked homogalcturonan (HG) epitope [8]
HG Backbone-2	JIM136	
	JIM7	Partially methyl esterified α-1,4 linked homogalcturonan (HG) epitope [8]
RG-I Backbone	CCRC-M69	[4)-α-D-GalpA-(1,2)-α-L-Rhap-(1,]$_n$ of RG-I backbone (Hahn, M.G, unpublished data)
	CCRC-M35	[4)-α-D-GalpA-(1,2)-α-L-Rhap-(1,] of RG-I backbone [9]
	CCRC-M36	[4)-α-D-GalpA-(1,2)-α-L-Rhap-(1,] of RG-I backbone (Hahn, M.G, unpublished data)
	CCRC-M14	[4)-α-D-GalpA-(1,2)-α-L-Rhap-(1,] of RG-I backbone (Hahn, M.G, unpublished data)
	CCRC-M129	[4)-α-D-GalpA-(1,2)-α-L-Rhap-(1,] of RG-I backbone (Hahn, M.G, unpublished data)
	CCRC-M72	[4)-α-D-GalpA-(1,2)-α-L-Rhap-(1,] of RG-I backbone (Hahn, M.G, unpublished data)
Linseed Mucilage	JIM3	

RG-I	CCRC-M40	
	CCRC-M161	
	CCRC-M164	
Physcomitrella Pectin	CCRC-M98	
	CCRC-M94	
RG-Ia	CCRC-M5	
	CCRC-M2	
RG-Ib	JIM137	
	JIM101	
	CCRC-M61	
	CCRC-M30	
RG-Ic	CCRC-M23	
	CCRC-M17	
	CCRC-M19	
	CCRC-M18	
	CCRC-M56	
	CCRC-M16	
RG-I/Arabinogalactan	CCRC-M60	
	CCRC-M41	
	CCRC-M80	
	CCRC-M79	
	CCRC-M44	
	CCRC-M33	
	CCRC-M32	
	CCRC-M13	
	CCRC-M42	
	CCRC-M24	
	CCRC-M12	
	CCRC-M7	β-(1,6)-Gal) DP>3 [10]
	CCRC-M77	
	CCRC-M25	
	CCRC-M9	

CCRC-M128
CCRC-M126
CCRC-M134
CCRC-M125
CCRC-M123
CCRC-M122
CCRC-M121
CCRC-M112
CCRC-M21
JIM131
CCRC-M22
JIM132
JIM1
CCRC-M15
CCRC-M8
JIM16

Arabinogalactan-1

JIM93
JIM94
JIM11
MAC204
JIM20

Arabinogalactan-2

JIM14
JIM19
JIM12
CCRC-M133
CCRC-M107

Arabinogalactan-3

JIM4
CCRC-M31
JIM17
CCRC-M26
JIM15
JIM8
CCRC-M85
CCRC-M81
MAC266

	PN 16.4B4
Arabinogalactan-4	MAC207<br>JIM133<br>JIM13<br>CCRC-M92<br>CCRC-M91<br>CCRC-M78
Unidentified	MAC265<br>CCRC-M97

References

1. Pattathil S, Avci U, Baldwin D, Swennes AG, McGill JA, Popper Z, Bootten T, Albert A, Davis RH, Chennareddy C *et al*: **A comprehensive toolkit of plant cell wall glycan-directed monoclonal antibodies**. *Plant Physiol* 2010, **153**(2):514-525.

2. Pattathil S, Avci U, Miller JS, Hahn MG: **Immunological approaches to plant cell wall and biomass characterization: Glycome Profiling**. *Methods Mol Biol* 2012, **908**:61-72.

3. Puhlmann J, Bucheli E, Swain MJ, Dunning N, Albersheim P, Darvill AG, Hahn MG: **Generation of monoclonal antibodies against plant cell-wall polysaccharides. I. Characterization of a monoclonal antibody to a terminal alpha-(1-->2)-linked fucosyl-containing epitope**. *Plant Physiol* 1994, **104**(2):699-710.

4. Schmidt D, Schuhmacher F, Geissner A, Seeberger PH, Pfrengle F: **Automated synthesis of arabinoxylan-oligosaccharides enables characterization of antibodies that recognize plant cell wall glycans**. *Chemistry* 2015, **21**(15):5709-5713.

5. Zhang X, Rogowski A, Zhao L, Hahn MG, Avci U, Knox JP, Glibert HJ: **Understanding how the complex molecular architecture of mannan-degrading hydrolases contributes to plant cell wall degradation.** *J Biol Chem* 2014, **289**:2002-2012.

6. Meikle PJ, Bonig I, Hoogenraad NJ, Clarke AE, Stone BA: **The location of (1-->3)-beta-glucans in the walls of pollen tubes of Nicotiana alata using a (1-->3)-beta-glucan-specific monoclonal antibody**. *Planta* 1991, **185**(1):1-8.

7. Meikle PJ, Hoogenraad NJ, Bonig I, Clarke AE, Stone BA: **A (1-->3,1-->4)-beta-glucan-specific monoclonal antibody and its use in the quantitation and immunocytochemical location of (1-->3,1-->4)-beta-glucans**. *Plant J* 1994, **5**(1):1-9.

8. Clausen MH, Willats WG, Knox JP: **Synthetic methyl hexagalacturonate hapten inhibitors of anti-homogalacturonan monoclonal antibodies LM7, JIM5 and JIM7**. *Carbohydr Res* 2003, **338**(17):1797-1800.

9. Young RE, McFarlane HE, Hahn MG, Western TL, Haughn GW, Samuels AL: **Analysis of the Golgi apparatus in Arabidopsis seed coat cells during polarized secretion of pectin-rich mucilage**. *Plant Cell* 2008, **20**(6):1623-1638.

10. Steffan W, Kovac P, Albersheim P, Darvill AG, Hahn MG: **Characterization of a monoclonal antibody that recognizes an arabinosylated (1-->6)-beta-D-galactan epitope in plant complex carbohydrates**. *Carbohydr Res* 1995, **275**(2):295-307.
